# Comparison of blood biochemical parameters of four species of vultures

**DOI:** 10.3897/BDJ.11.e97164

**Published:** 2023-01-20

**Authors:** Rusko Petrov, Dobri Yarkov, Tsvetan Chaprazov, Yana Andonova, Stefka Dimitrova, Ivanka Lazarova

**Affiliations:** 1 Green Balkans - Stara Zagora NGO, Stara Zagora, Bulgaria Green Balkans - Stara Zagora NGO Stara Zagora Bulgaria; 2 Trakia University, Stara Zagora, Bulgaria Trakia University Stara Zagora Bulgaria

**Keywords:** Bearded Vulture, Cinereous Vulture, Egyptian Vulture, Griffon Vulture, scavenger birds, biodiversity

## Abstract

Vultures play a very important role in ecosystems by feeding on dead animals and preventing the spread of pathogens. In the mid-20^th^ century in Bulgaria, all species of vultures experienced a rapid population decline and conservation measures include captive breeding and release via adaptation aviaries. Knowledge of the baseline blood biochemical parameters is crucial for the care, rehabilitation and prior to the release of endangered birds of prey. Plasma levels provide valuable information for the evaluation of the physical condition of animals.

Between 2020 and 2022, we took blood samples from captive Bearded, Griffon, Cinereous and Egyptian Vultures in Bulgaria (n = 118). We determined the values of 18 parameters - alanine transaminase, albumin, alkaline phosphatase, amylase, aspartate transaminase, calcium, chloride, cholesterol, creatine kinase, creatinine, glucose, lactate dehydrogenase, magnesium, phosphorus, total bilirubin, total protein, triglycerides and uric acid. This research determined the mean blood biochemical indices for aviary Bearded, Griffon, Cinereous and Egyptian Vultures in Bulgaria and compared the values amongst the four vulture species, to serve in determining clinical pathology and nutrition for scavenger birds of different species, age groups and genders in the country.

The results of this study suggested that there are significant differences between many of the indicators of the four vulture species. There are fewer differences in the indicators of different ages of birds of a given species and almost no differences are found between the two sexes of a species. These values could be used by scientists, veterinary pathologists, wildlife rehabilitation centres and other researchers. Furthermore, the use of such parameters in assessing population health may enable conservationists to further research environmental conditions affecting the vultures’ reproduction and survival.

## Introduction

Through their feeding habits, vultures play a very important role in the ecosystem’s balance as they prevent the spread of pathogens (DeVault et al. 2016). All vulture species in Bulgaria experienced a massive population decline starting from the mid-20^th^ century (Arabadzhiev 1962). Currently, the Bearded Vulture (*Gypaetusbarbatus*) is marked as extinct in the Red Data Book of the Republic of Bulgaria (Boev 2015) and the only birds of this species in the country can be found in the Wildlife Rehabilitation and Breeding Centre (WRBC) - Green Balkans, part of Green Balkans - Stara Zagora NGO. The Cinereous Vulture (*Aegypiusmonachus*) was marked as Extinct in the Red Data Book of the Republic of Bulgaria (Marin et al. 2015). Its population saw a rise after years of conservation and re-introduction efforts - moving on from the last confirmed breeding of a single pair in 1993 (Marin et al. 1998) and, as of 2021, there are six pairs nesting in the wild in the country, formed of re-introduced birds (Ivanov et al. 2022). The Egyptian Vulture (*Neophronpercnopterus*) population in Bulgaria is currently in decline - it decreased by more than 50% between 2003 and 2016 (Arkumarev et al. 2018). The species is marked as "Endangered" both in the Red Data Book of the Republic of Bulgaria (Kurtev et al. 2015) and in the IUCN Red List (BirdLife International 2021). As a result of the targeted efforts of Bulgarian nature conservation NGOs for biodiversity preservation (Nikolova 2010), the nesting Griffon Vulture (*Gypsfulvus*) population recovered from one colony in the 1970s (Michev et al. 1980) to 100 pairs in 13 colonies in 2016 (Stoynov et al. 2018). In the Red Data Book of the Republic of Bulgaria, its status is "Endangered" (Yankov et al. 2015).

Keeping vultures in breeding or adaptation aviaries requires veterinary care. Plasma levels of some biochemical indices provided valuable information for the evaluation of the physical condition and nutritional status of raptors (Garcia-Rodriguez et al. 1987, Ferrer 1993, Dawson and Bortolotti 1997, Ferrer and Dobado-Berrios 1998, Baumbusch et al. 2021). Their feeding cycles and body condition determined the urea levels, pH of the urine, total protein level, cholesterol and blood glucose levels, which can be used to optimise the feeding of the birds in conditions with limited space and movement opportunities. Vulture blood indicators values may be affected by factors such as species, sex, age and diet (Dell'omo and Cavallina 1996, Villegas et al. 2002, Hernández and Margalida 2010). Values differed between wild populations and vultures which have undergone prolonged captivity (Dobado-Berrios et al. 1998, Giambelluca et al. 2017). Knowledge of the baseline blood values of captive birds is needed in order to be able to assess individuals from captive breeding programmes (Petrak 1982, Polo et al. 1992). It is extremely relevant for endangered species and part of re-introduction programmes. For the Bearded Vulture, there has been no previous study of the plasma parameters of captive individuals and the values of the four European vultures species have not yet been compared.

The goal of this research was to determine the baseline blood biochemical indices in captive Bearded, Cinereous, Egyptian and Griffon Vultures in Bulgaria, to serve in determining clinical pathology, nutrition and veterinary management and be able to provide more adequate care for them as part of the work to stabilise their populations in the country.

## Material and methods

All four vulture species are breeding in captivity in Bulgaria and three (Cinereous, Egyptian and Griffon) have been kept and released from adaptation aviaries into the wild in the country. The birds were sampled either in the adaptation aviaries or in the WRBC if they were part of the breeding pairs there. The birds in the aviaries have undergone either short- or long-term captivity, based on when and if they were there for release. Sampled were seven Bearded, 40 Cinereous, 21 Egyptian and 50 Griffon Vultures.

All birds were examined by a veterinary physician of the WRBC upon blood collection and were determined to be clinically healthy. Surfaces were disinfected with a Desclean solution. We disinfected the area and collected 1.5 ml of whole blood from either the left or right basilic vein (*Vena cutanea ulnaris superficialis*) of all specimens tested. We immediately placed the blood into collection tubes containing lithium heparin. We used 3 ml syringes with 23G needles. We transported the samples to a laboratory, where they were processed within 4 hr of collection using a BS-120 (Mindray, China) automatic biochemical analyser.

The age of the Bearded Vultures was divided into: juvenile - covering the period up to the age of 1 year , immature - 1-3 years-old, subadult - 4-5 years-old and adult > 6 years-old (Heredia and Margalida 2022). The Cinereous Vulture age groups were adapted from De la Puente and Elorriaga (2012) into: - juvenille - 1 years-old, immature - 1-3 years-old, subadult - 4-5 years-old and adult > 6 years-old. The age of the Egyptian Vultures was divided into: juvenile - up to 1 years-old, immature - 1-2 years-old, subadult - 2-3 years-olds and adult - > 4 years-old (Blasco-Zumeta and Hainze 2013b). The age of Griffon Vultures was set as: juvenile - 1 years-old; immature - 1-3-years-old; subadult - 4-5 years-old and adult - > 5-years-old (Blasco-Zumeta and Hainze 2013a).

We compared the values of the following 18 biochemical parameters - alanine transaminase (ALT, U/I), albumin (g/l), alkaline phosphatase (ALP, U/I), amylase (U/I), aspartate transaminase (AST, U/I), calcium (mmol/l), chloride (mmol/l), cholesterol (mmol/l), creatine kinase (CK, U/I), creatinine (μmol/l), blood glucose (mmol/l), lactate dehydrogenase (LDH, U/I), magnesium (mmol/l), phosphorus (mmol/l), total bilirubin (μmol/l), total protein (g/l), triglycerides (TG, mmol/l) and uric acid (μmol/l), using descriptive statistics with compared means (n) and Standard Deviation (SD) using analyses of variance (one way ANOVA) provided in SPSS Statistics (SPSS-Inc., 2019, Chicago, USA). Differences were considered significant at p < 0.05.

## Results

We determined the mean values of the four species of European vultures residing in captivity in Bulgaria. We compared the values of the 18 biochemical parameters amongst the four species - there was a statistically significant difference in 10 parameters - AST, ALT, LDH, CK, cholesterol, TG, amylase, calcium, albumin and creatinine. Eight were not significantly different (Table 1).

A detailed analysis of the factor Sex on the biochemical indicators showed that it does not affect the average values of the four species of birds. An exception is the Cinereous Vultures where there is a moderate statistical difference in calcium and phosphorus between males and females (F = 5.55; p = 0.024). A statistically significant difference in the Age factor was found on three biochemical indicators of the Griffon Vultures - ALP, cholesterol and phosphorus (F = 14.69; p = 0.00; F = 7.20; p = 0.00; F = 2.91; p = 0.04, respectively). In Cinereous Vultures, the factor Age affected significantly some biochemical indices such as ASAT, LDH, CK, TG, chloride, calcium, phosphorus, total protein, albumin, blood glucose and uric acid. In the Bearded Vultures, the biochemical indices ALP, CK, cholesterol and chloride were significantly different. A similar tendency was also found in the Egyptian Vultures in the parameters ALP, calcium, total protein, total bilirubin and uric acid (Table 2).

## Discussion

We compared our results to previously published biochemistry values for vultures. For the Bearded Vulture, there is only one study available in which wild birds were sampled (Hernández and Margalida 2010). Compared to their values, ours of the captive birds were noticeably higher for the parameters ALP, amylase and uric acid and lower for LDH and CK. However, when determining our mean values, we combined age groups, but we compared them with the published results for only adult birds. For the Cinereous Vultures, we compared our data to two studies. We have taken the results of captive birds (Villegas et al. 2002), as we assumed they would be closest to ours. Additionally, we also looked at another study featuring the values of wild birds (Seok et al. 2017). Our biochemical indices AST, ALP, LDH and CK values were higher than those of the two studies and theirs had a low protein level. The uric acid value for our Cinereous Vultures was much closer to that of the wild birds studied and not to those of the captive group. The results of the Egyptian Vultures were compared to three other studies on the birds’ biochemistry (Polo et al. 1992, Dell'omo and Cavallina 1996, Dobado-Berrios et al. 1998). The values of captive birds were compared. AST, ALT, ALP, LDH and CK were higher in our vultures than in most of the rest. Our CK result was of similar value to the research of Dell'omo and Cavallina (1996). Our Griffon Vulture results were compared to other studies of captive birds (Ferrer et al. 1987, Polo et al. 1992) and the results were comparable, with the exception of parameters AST, ALT, ALP, LDH and CK - which, in our birds, are with considerably higher values again. The variations could be due to different diets and physical activity in the different captive facilities. It is reported the elevated AST and ALT values in particular could be attributed to lower physical activity associated with confinement (Dobado-Berrios et al. 1998).

This is the first study in which the four European vulture species were sampled in one country in a short period of time and compared. In between them, there was a statistically significant difference found in 10 biochemical parameters, while there was no significant difference in eight. It has been reported there are differences in blood chemistry values between wild and captive vultures (Dobado-Berrios et al. 1998, Villegas et al. 2002). Giambelluca et al. (2017) have suggested that length of captivity can affect certain (haematological) parameters of vultures - birds that had undergone short-term captivity (< 30 days) exhibited values closer to that of wild birds. In this regard, we considered the birds in this study captive, as all had spent more than a month in aviaries.

The factor Sex did not affect significantly any of the average values of the four species, which was also determined in some of the above-mentioned studies (Dell'omo and Cavallina 1996, Hernández and Margalida 2010). Age-related differences were observed in a number of indicators for all four species, which is consistent with findings for Bearded Vultures by Hernández and Margalida (2010), for Cinereous Vultures by Villegas et al. (2002) and for Egyptian Vultures (Dell'omo and Cavallina 1996, Dobado-Berrios et al. 1998).

## Conclusions

This is the first study in which the four European vulture species kept in captivity were sampled and the values of some biochemical parameters were compared. A number of differences were found amongst the biochemical indicators of the four species, indicating that the values for one species should not be used for another. There were no significant differences found between males and females of the four species. Age-related differences were observed in a number of indicators for all four vulture species, indicating values for one age group are not directly applicable to another. Compared to previously published research on European vulture biochemistry, the values of AST, ALT, ALP, LDH and CK were significantly higher in the birds in this study, which can be attributed to variations in diet and physical activity in the different facilities. The reported values can be used for assessing the vultures’ health as part of the work to stabilise their populations in Bulgaria.

## Figures and Tables

**Table 1.** Average values of some biochemical parameters of vultures in Bulgaria (Mean ± Standard Deviation, n = number).

	Bearded Vulture (n = 7)	Cinereous Vulture (n = 40)	Egyptian Vulture (n = 21)	Griffon Vulture (n = 50)	All (n = 118)	
	Mean ± SD	Mean ± SD	Mean ± SD	Mean ± SD	F	Sig.
AST, U/I	177 ± 19.06	338.95 ± 16.74	290.9 ± 27.30	330.48 ± 15.33	5.09	0.00
ALT, U/I	17.28 ± 2.88	48.8 ± 2.99	30.23 ± 1.93	50.68 ± 2.89	12.65	0.00
ALP, U/I	277.42 ± 174.80	287.25 ± 37.23	354.95 ± 75.21	409.64 ± 89.09	0.56	0.64
LDH, U/I	783.57 ± 104.73	1132.12 ± 85.85	1109.66 ± 132.99	725.92 ± 48.51	6.86	0.00
CK, U/I	556.85 ± 259.29	999.65 ± 134.27	1014.9 ± 138.27	605.82 ± 84.52	3.26	0.02
Cholesterol, mmol/l	6.44 ± 0.30	5.23 ± 0.12	6.19 ± 0.25	5.35 ± 0.24	3.73	0.01
TG, mmol/l	0.75 ± 0.10	0.68 ± 0.03	1.63 ± 0.10	0.8 ± 0.04	46.97	0.00
Chloride, mmol/l	111.71 ± 1.75	112.5 ± 0.75	112.76 ± 1.04	110.92 ± 0.72	1.07	0.37
Amylase, U/I	1699.71 ± 267.27	1177.7 ± 87.29	839.19 ± 59.88	1894.04 ± 190.36	7.62	0.00
Calcium, mmol/l	2.66 ± 0.05	2.63 ± 0.06	2.24 ± 0.03	2.62 ± 0.04	8.58	0.00
Phosphorus, mmol/l	1.03 ± 0.12	1.03 ± 0.08	1 ± 0.08	1.25 ± 0.11	1.25	0.29
Magnesium, mmol/l	0.92 ± 0.03	0.91 ± 0.02	0.85 ± 0.04	0.96 ± 0.03	1.92	0.13
Total protein, g/l	41.12 ± 4.18	46.13 ±1 .04	44.63 ± 1.22	44.02 ± 1.16	1.15	0.33
Glucose, mmol/l	15.58 ± 0.39	17.15 ± 0.39	13.85 ± 0.59	15.69 ± 0.44	0.54	0.66
Albumin, g/l	16.87 ± 0.33	17.73 ± 0.45	16.91 ± 0.19	17.5 ± 0.44	6.66	0.00
Total bilirubin, μmol/l	5.61 ± 0.95	9.88 ± 0.67	9.39 ± 1.66	9.58 ± 0.62	1.47	0.23
Creatinine, μmol/l	38.28 ± 1.72	45.3 ± 0.68	35.9 ± 0.74	42.6 ± 0.90	16.36	0.00
Uric acid, μmol/l	484.14 ± 74.60	380.35 ± 21.56	369.57 ± 64.82	468.74 ± 41.24	1.45	0.23

**Table 2.** Significance of factors Sex and Age on the biochemical values of Griffon Vulture, Cinereous Vulture, Breaded vulture and Egyptian Vulture (n = number). Differences were considered significant at p < 0.05.

		Bearded Vulture (n = 7)		Cinereous Vulture (n = 40)		Egyptian Vulture (n = 21)		Griffon Vulture (n = 50)	
		F	Sig.	F	Sig.	F	Sig.	F	Sig.
AST, U/I	Sex	0.33	0.59	1.98	0.17	0.72	0.41	1.39	0.24
	Age	0.34	0.80	4.12	0.01	2.31	0.11	1.58	0.21
ALT, U/I	Sex	4.51	0.09	0.94	0.34	1.38	0.26	0.01	0.92
	Age	0.96	0.51	1.42	0.25	1.08	0.38	1.59	0.20
ALP, U/I	Sex	1.11	0.34	0.01	0.94	2.14	0.16	0.29	0.59
	Age	49.89	0.00	2.47	0.08	4.40	0.02	14.69	0.00
LDH, U/I	Sex	0.44	0.53	2.72	0.11	1.61	0.22	0.00	0.95
	Age	0.89	0.54	3.34	0.03	0.70	0.56	0.30	0.82
CK, U/I	Sex	0.67	0.45	1.54	0.22	0.28	0.60	0.01	0.91
	Age	25.12	0.01	7.75	0.00	0.94	0.44	0.34	0.80
Cholesterol, mmol/l	Sex	0.03	0.86	0.78	0.38	0.91	0.35	0.11	0.74
	Age	11.44	0.04	2.04	0.13	1.99	0.15	7.20	0.00
TG, mmol/l	Sex	0.59	0.48	1.77	0.19	0.42	0.52	2.47	0.12
	Age	0.55	0.68	3.27	0.03	0.87	0.48	2.55	0.07
Chloride, mmol/l	Sex	0.68	0.45	0.69	0.41	0.32	0.58	3.28	0.08
	Age	63.71	0.00	5.08	0.00	2.27	0.12	0.27	0.85
Amylase, U/I	Sex	1.50	0.28	0.04	0.85	0.06	0.81	0.00	0.96
	Age	0.38	0.77	1.14	0.35	3.14	0.05	1.36	0.27
Calcium, mmol/l	Sex	0.27	0.62	5.55	0.02	1.11	0.31	1.40	0.24
	Age	3.08	0.19	4.85	0.01	9.59	0.00	0.68	0.57
Phosphorus, mmol/l	Sex	3.08	0.14	4.63	0.04	1.23	0.28	1.08	0.30
	Age	1.48	0.38	3.56	0.02	1.83	0.18	2.91	0.04
Magnesium, mmol/l	Sex	0.86	0.40	2.24	0.14	0.13	0.72	2.17	0.15
	Age	0.04	0.99	0.49	0.69	0.36	0.78	0.43	0.74
Total protein, g/l	Sex	0.35	0.58	0.69	0.41	0.02	0.89	0.07	0.80
	Age	0.96	0.51	8.16	0.00	11.23	0.00	1.08	0.37
Albumin, g/l	Sex	0.74	0.43	0.71	0.41	0.13	0.72	0.35	0.56
	Age	0.05	0.98	13.38	0.00	0.98	0.42	0.76	0.52
Glucose, mmol/l	Sex	0.25	0.64	1.79	0.19	0.21	0.65	0.83	0.37
	Age	4.84	0.11	4.35	0.01	1.68	0.21	0.12	0.95
Total bilirubin, μmol/l	Sex	4.56	0.09	1.33	0.26	0.69	0.42	0.25	0.62
	Age	4.36	0.13	1.66	0.19	4.68	0.01	2.35	0.08
Creatinine, μmol/l	Sex	0.43	0.54	0.13	0.72	0.17	0.68	0.02	0.89
	Age	4.02	0.14	1.21	0.32	1.59	0.23	2.40	0.08
Uric acid, μmol/l	Sex	0.00	0.98	0.32	0.57	0.12	0.73	0.28	0.60
	Age	0.52	0.70	4.95	0.01	40.22	0.00	2.35	0.09
